# European expert network on rare communicable diseases and other rare diseases linked to mobility and globalisation focused on health care provision (EURaDMoG): a feasibility study

**DOI:** 10.1186/s13023-020-01534-1

**Published:** 2020-10-16

**Authors:** Ana Requena-Méndez, Zeno Bisoffi, Joan-Lluis Vives-Corrons, Joaquim Gascon, Antoni Plasència

**Affiliations:** 1 Barcelona Institute for Global Health (Hospital Clínic- Universitat de Barcelona), Barcelona, Spain; 2 Department of Medicine, Karolinska Institutet, Solna, 17176 Stockholm, Sweden; 3 Department of Infectious - Tropical Diseases and Microbiology, IRCCS Sacro Cuore Don Calabria Hospital, Negrar (Verona), Italy; 4 Department of Diagnostics and Public Health, University of Verona, Verona, Italy; 5 Institute for Leukaemia Research Josep Carreras (IJC), Badalona (Barcelona), Spain

**Keywords:** Rare diseases, Mobility, Globalisation, Imported diseases, Communicable diseases, Rare infections

## Abstract

**Introduction:**

In the current mobility and globalization context, there is a growing need to identify potential changes on the pattern of diseases in the European Union (EU)/European Economic Area (EEA) and provide accurate diagnosis and treatment for the population. The pattern of rare communicable diseases that can affect people returning to EU/EEA from travel abroad, visiting EU/EEA or establishing in the EU/EEA is of special relevance. The objective of this manuscript is to give an overview about the EURaDMoG study and discuss the feasibility of establishing a European network on rare communicable diseases and other rare conditions linked to mobility and globalization.

**Methods:**

We undertook a three-steps process where we first conducted a narrative review to estimate the prevalence and incidence and to list rare communicable and non-communicable diseases linked to mobility and globalization in the EU/EEA; second, we organized an international consultation workshop with experts in the diseases previously selected; and finally, the feasibility study analysed how successful a European expert network on rare diseases linked to mobility and globalization focused on health care provision would be, accounting for different operational and also sustainability criteria.

**Results:**

First, considering the areas or topics that the network should cover, it was concluded that communicable and non-communicable rare diseases linked to mobility and globalization should be differentiated. Second, since all non-communicable rare diseases linked to mobility and globalization identified are already covered by different European Reference Networks (ERNs), there is no need for them to be included in a new European network. Three scenarios were considered for establishing a potential European network for rare communicable diseases linked to Mobility and Globalisation with a focus on Health Care provision: 1) To maintain the current situation “Status Quo” scenario; 2) to create a specific European expert network (EEN) on rare communicable diseases linked to mobility and globalisation; 3) to develop a new ERN on communicable rare diseases linked to mobility and globalisation.

**Conclusions:**

Since the focus is the provision of health care, an ERN could have the potential to better boost the quality of care being facilitated by technological tools and online platforms that permit the safe and ethically acceptable exchange of data. However, this potential new network should not eclipse current existing networks and they should be complementary.

## Background

Population movements have had a major impact on disease epidemiology and public health [1]. There is a growing need to provide health care for “rare” diseases that are related to this context. The European Union (EU) /European Economic Area (EEA) countries are tackling new emerging diseases not endemic in EU countries [2] which are not considered rare diseases at the world-wide level, but which are rare conditions in Europe according to the definition of rare diseases from the European Commission (EC) [3] or are emerging due to climate change.

Such a definition of rare diseases was adopted by the Community Action Programme on rare diseases 1999–2003 as those life-threatening or chronically debilitating diseases presenting a prevalence less than 5 per 10,000 people in the EU [4].

In addition to the limited number of patients, rare diseases are characterised by by scarce knowledge and expertise, single them out as a distinctive domain of very high European added value [5]. European cooperation can help to ensure that scarce knowledge can be shared and resources combined as efficiently as possible, in order to tackle rare diseases effectively across the EU/EEA as a whole [4]. Classically, most rare diseases are genetic disorders, that is, non-infectious inherited conditions [6], but there are also other rare diseases like rare cancers, auto-immune diseases, toxic disorders and communicable diseases. Imported diseases which are not familiar to European health professionals, have recently been recognized as a new challenge as they have been found to be prevalent only in mobile populations coming from tropical countries [7]. Although most migrants and newly arriving refugees do not pose a special challenge for the autochthonous population in terms of communicable diseases risk [8], they are disproportionately affected by infectious diseases that at some point may have an impact on the health system (critical care for immunosuppressed migrant patients, transplant programs ...) [9]. Therefore, the risk of communicable diseases (CD) is in accordance to the country of origin and/or transit of migrants and refugees because prevalence rates and burden of CD differ considerably by country [10]. In this regard, additional work is needed to improve the knowledge gaps among different categories of health professionals [9].

In the same way, the increase of global mobility related to tourism or trade has increased the risks of rare communicable diseases in travellers, particularly vector borne diseases [11]. During the last century, invasive mosquito species have become widely established across Europe [12]. Although subsequent transmission and outbreaks of unfamiliar diseases such as Chikungunya and Dengue are in part due to increased globalisation, with intercontinental air travel and global shipping transport [11], changes in vector distributions are also being driven by climatic changes and changes in land use, infrastructure, and the environment [13].

Regarding rare CD, health professionals often lack the necessary skills to correctly identify and treat unfamiliar pathologies, due to their low prevalence in the countries where they are working or because they are newly emerging diseases [14]. In the same way, the increase in global mobility for tourism or trade poses similar risks of rare diseases in travellers, often implying a particular challenge for diagnosis and management [11]. It is also worth mentioning the effect of climate change on vector borne diseases [15]. Sharing medical knowledge and expertise is an important strategy for developing the competencies and skills of health professionals to address patients’ needs and support change in health service provision [16, 17]. It is clear that the focus here is the diagnosis and treatment, not the epidemiological surveillance of the diseases.

On the other hand, non-communicable diseases (NCD) may also represent a challenge in mobile populations, particularly in migrants. Haemoglobin disorders such as thalassemia or sickle cell disease (SCD) are inherited disorders whose frequency varies by ethnic group [18]. Due to population movements, the sickle cell trait has been spread to other places such as the Middle East and Southern Europe [19], where thalassemia has also been endemic until now, also due to the historical endemic presence of malaria until recently. Most rare non communicable diseases are already covered by the existing ERN [20].

The objective of this study was to evaluate the feasibility of a European expert network on rare diseases linked to mobility and globalization focused on Health Care provision.

## The EURaDMoG feasibility study

This study analysed how successfully a European expert network on rare diseases linked to mobility and globalization focused on Health Care provision would be, accounting for different factors or criteria that will be outlined. Through this study, potential positive and negative outcomes of the network have been envisaged. All these factors have been separately assessed considering different scenarios and have been evaluated based on the results of a consultation workshop, with a combined three-step approach: [1] conducting a comprehensive review, a [2] consultation workshop, [3] an overall feasibility assessment. The main results are described hereunder.

1. A comprehensive review was carried out to assess epidemiological, diagnostic and treatment aspects of rare diseases (both communicable and non-communicable) linked to Mobility and Globalisation and to elaborate a list of rare diseases linked to mobility and globalisation.

The primary objective was to assess which rare diseases (according to the EC definition of rare disease) should be covered by a potential expert European network of rare disease linked to mobility and Globalisation, to review their prevalence and/or incidence in EU/EEA countries as well as their epidemiological profile.

For that purpose, first a narrative review of the literature has been conducted to explore which rare diseases (both communicable and non-communicable) in EU/EEA countries are linked to mobility and Globalization. For communicable diseases, first, we reviewed the Orphanet list if rare disease [21], selecting those that are associated with mobility and globalisation. This relationship was established either because these diseases are more frequent in mobile populations (migrants and travellers) irrespective of whether they are imported diseases or not, or because of the risk of being introduced /reintroduced or of changing their epidemiology in EU/EEA countries due to environmental factors such as climate change. We also conducted an online survey with experts in CD to agree the preliminary of CD identified. The survey was distributed among European network targeting most of the diseases identified in the list The European Network for Tropical Medicine and Travel Health (TROPNET) [22], the European Travel and Tropical Medicine Network (EUROTRAVNET) [23] and the European Expert laboratory network for emerging viral diseases (EVDLABNET) [24].

Thereafter, the prevalence /incidence of these disorders were comprehensively reviewed through a narrative review of the literature [25]. Concerning CD, we have identified more than 130 infections that could be considered “rare diseases” in EU/EEA countries and that are linked to mobility and globalisation (See Table 1).. In addition, many studies also highlight that most of these diseases do not have appropriate and widely available diagnostic techniques and treatments.

**Table 1:** List of rare communicable diseases linked to mobility and globalisation identified throughout the euradmog study

DISEASE related keywords	MICROORGANISM related keywords
**BACTERIAL INFECTIONS**	
Anaplasmosis	*Anaplasma phagocytophilum*
Ehrlichiosis	*Ehrlichia* spp.
Endemic typhus, Murine typhus /flea-borne typhus	*Rickettsia typhi*
Epidemic typhus	*Rickettsia prowazekii*
Mediterranean spotted fever	*Rickettsia conorii*
Rickettsialpox	*Rickettsia akari*
Scrub typhus, Tsutsugamushi disease	*Orientia tsutsugamushi*
Rocky mountain spotted fever	*Rickettsia rickettsii*
African tick typhus	*Rickettsia africae*
Relapsing fever	*Borrelia recurrentis*
Lyme disease	*Borrelia burgdoferi*
Other rickettsiosis	*Rickettsia* spp.
Anthrax	*Bacillus anthracis*
Tetanus	*Clostridium tetani*
Trachoma	*Chlamydia trachomatis*
Chancroid	*Haemophilus ducreyi*
Granuloma inguinale- Donovanosis	*Klebsiella granulomatis*
Actinomycosis	*Actinomyces israelii*
Treponema infections Yaws	*Treponema pallidum pertenue*
Buruli ulcer	*Mycobacterium ulcerans*
Hansen’s disease / Leprosy	*Mycobacterium leprae*
Leptospirosis	*Leptospira interrogans*
Bartonellosis, Oroya fever, Carrion disease	*Bartonella bacilliformis*
Bartonellosis, Trenchs fever	*Bartonella quintana*
Brucellosis, Malta fever	*Brucella* spp*.*
Melioidosis	*Burkholderia pseudomallei*
Paratyphoid fever	*Salmonella enterica* serotype Paratyphi
Typhoid fever	*Salmonella enterica* serotype Typhi
Rheumatic fever	*Streptococcus pyogenes*
Q fever, Nine mile fever, Quadrilateral fever, Query fever	*Coxiella burnetii*
Secondary non-tropical sprue - Whipple disease	*Tropheryma whipplei*
Cholera	*Vibrio cholerae*
Botulism	*Clostridium botulinum*
Tropical pyomyositis*---*	
Tularemia	*Francisella tularensis*
Plague	*Yersinia pestis*
Diphtheria	*Corynebacterium diphtheriae*
**FUNGAL INFECTIONS**	
Chromomycosis/Chromoblastomycosis	*Fonsecaea pedrosoi, Phialophora verrucosa and Cladophialophora carrionii*
Madura foot /Eumycetoma	*Madurella mycetomatis*
Sporotrichosis	*Sporothrix schenckii*
Paracoccidioidomycosis	*Paracoccidioides brasiliensis*
Coccidioidomycosis, Desert fever, San Joaquin valley fever, California disease	*Coccidioides immitis*
Peniciliosis	*Penicillium marneffei*
Scedosporiosis	*Scedosporium* spp.
**HELMINTH INFECTIONS**	
Cystic echinococcosis, Echinococcosis	*Echinococcus granulosus*
Alveolar echinococcosis	*Echinococcus multilocularis*
Ancylostomiasis /Ankylostomiasis	*Ancylostoma duodenale /Necator americanus*
Angiostrongyliasis	*Angiostrongylus cantonensis*
Strongyloidiasis, Anguilluliasis	*Strongyloides stercoralis*
Anisakiasis	*Anisakis* spp.
Ascariasis	*Ascaris lumbricoides*
Bilharziasis, Schistosomiasis	*Schistosoma* spp.
Clonorchiasis	*Clonorchis sinensis*
Opisthorchiasis	*Opisthorchis viverrini*
Cutaneous larva migrans	*Ancylostoma* spp*.*
Cysticercosis	*Taenia solium*
Diphyllobothriasis	*Diphyllobothrium latum*
Dirofilariasis	*Dirofilaria immitis*
Distomatosis	*Other trematodes-Heterophyes heterophyes, Metagonimus* spp.
Dracunculiasis, Guinea Worm disease	*Dracunculus medinensis*
Fascioliasis	*Fasciola* spp.
Paragonimiasis	*Paragonimus* spp.
Gnathostomiasis	*Gnathostoma* spp.
Hymenolepiasis	*Hymenolepsis nana*
Loiasis	*Loa loa*
Lymphatic filariasis	*Wuchereria bancrofti / Brugia malayi*
Mansonelliasis	*Mansonella* spp.
Onchocerciasis	*Onchocerca volvulus*
Sparganosis	*Spirometra* spp.
Taeniasis	*Taenia* spp.
Trichinosis	*Trichinella spiralis*
Trichuriasis	*Trichuris trichiura*
**PROTOZOAL INFECTIONS**	
Acanthamoeba infection, Keratitis	*Acanthamoeba* spp.
African trypanosomiasis, Sleeping sickness	*Trypanosoma brucei*
Amebiasis	*Entamoeba histolytica*
Amebic meningoencephalitis	*Naegleria fowleri*
American trypanosomiasis, Chagas disease	*Trypanosoma cruzi*
Babesiosis	*Babesia* spp.
Cryptosporidiosis	*Cryptosporidium parvum*
Cyclosporiasis	*Cyclospora cayetanensis*
Isosporiasis	*Isospora belli*
Leishmaniasis	*Leishmania* spp.
Malaria	*Plasmodium* spp.
Sarcocystosis, Sarcosporidiosis	*Sarcocystis hominis*
**VIRAL INFECTIONS**	
Avian flu	Avian influenza
Brazilian haemorrhagic fever	Sabia virus
California encephalitis	California encephalitis virus
Chapare haemorrhagic fever	Chapare virus
Chikungunya	Chikungunya virus
Colorado tick-borne disease, Mountain fever, American mountain fever, Mountain tick fever-	Colorado tick fever (CTF)
Crimea-Congo haemorrhagic fever	CCHF virus
Dengue fever	Dengue virus
Ebola	Ebola virus
Hantavirosis, Haemorrhagic fever-renal syndrome	Hantavirus
Hepatitis D	Hepatitis D virus
Hepatitis E	Hepatitis E virus
Herpes B infection, B virus infection	Herpesvirus simiae, monkey B virus
Tropical spastic paraparesis	Human T-lymphotropic virus 1 (HTLV-1)
Japanese encephalitis	Japanese encephalitis virus (JEV)
Junin haemorrhagic fever, Argentine haemorrhagic fever	Junin virus
Kyasanur haemorrhagic fever, Kyasanur forest disease, Monkey fever, Monkey disease	KFD virus (KFDV)
La Crosse encephalitis	La Crosse virus (LACV)
Lassa haemorrhagic fever	Lassa virus
Lujo haemorrhagic fever, Zambian haemorrhagic fever	Lujo virus
Machupo haemorrhagic fever	Machupo virus (MACV)
Marburg haemorrhagic fever /Marburg virus disease	Marburg virus
Nipah encephalitis, Nipah fever /Nipah virus disease	Nipah virus
Omsk haemorrhagic fever	Omsk haemorrhagic fever virus (OHFV)
Poliomyelitis, Poliomyelitis in patients with immunodeficiency deemed at risk	Poliovirus
Rabies	Rabies virus
Rift valley fever	Rift valley fever (RVF) virus
Saint Louis encephalitis	Saint Louis encephalitis (SLE) virus
Tick-borne encephalitis	Tick-borne encephalitis (TBE) virus
Venezuelan haemorrhagic fever	Guanarito virus
Western equine encephalitis, Western equine encephalomyelitis	Western equine encephalitis (WEE) virus
West-Nile encephalitis, West-Nile fever	West-Nile virus (WNV)
Yellow fever	Yellow fever virus
Zika virus disease	Zika virus
Middle East respiratory syndrome coronavirus	MERS-CoV
**OTHER CONDITIONS**	
Tick paralysis	Tick
Cutaneous myiasis	*Dermatobia hominis, Cordylobia anthropophaga*
African iron overload	
Ciguatera fish poisoning	
Tropical calcific chronic pancreatitis	Tropical pancreatitis
Tropical pancreatic diabetes	
Tropical endomyocardial fibrosis (TEF)	
Acquired Creutzfeldt-Jakob disease	prion disease
Hyperreactive malarial splenomegaly	
Genital female mutilation	
Severe fever with thrombocytopenia syndrome	
**INFECTIONS WITH PARTICULAR ASPECTS TO BE CONSIDERED**	
Giardiasis	*Giardia duodenalis*
Shiga-like toxin-associated HUS	*E.coli* (O157)
Tuberculosis	*Mycobacterium tuberculosis*

Concerning NCD, we have comprehensively reviewed the literature to assess the association between traditional rare diseases (non CD) with mobility and globalization in EU/EEA countries [25]. The online survey was also conducted with experts belonging to the 24 European Reference Networks (ERN) in the EU/EEA to agree the preliminary of rare non-CD identified. Some autosomal recessive disorders have been particularly found to be associated with mobility and globalization, in part because co-sanguinity is more frequent in certain migrant populations.

Other results of the review also showed that most studies were conducted in highly specialized units (particularly in Tropical diseases), suggesting a lack of knowledge among other health professionals regarding imported diseases. It was also concluded that the areas of expertise are quite different between rare NCD and CD. In the particular case of CD, a high expertise is required in some areas of infectious disease, particularly in Tropical Medicine, imported diseases and also tick-borne diseases. Due to changes in the epidemiology of rare CD (e.g. West Nile virus or Chikungunya), a network including experts from countries where these diseases are less rare would be helpful to clinicians working in countries where cases are rarely diagnosed. In this regard, although a list of CD has been proposed (See Additional file 1), the list should remain open to be updated in case new diseases are emerging. All these challenges should be considered when designing appropriate strategies to improve the health care provision related to these diseases in people living in EU/EEA countries.

2. A consultation Workshop was conducted on the “Assessment of a European Expert Network on communicable diseases and other rare pathologies in the context of Mobility and Globalization”. Participants of the online survey and belonging to the networks TROPNET, EUROTRAVNET, EVDLABNET and the existing 24 ERN in the EU/EEA were invited to participate to have a minimum of two health care professionals per country in the EU (the countries participating the study) with expertise on rare diseases linked to mobility and globalisation. Whenever possible, they were selected as one member with expertise in rare-CD (imported or vector-borne diseases) and the other one with expertise in rare NCD. The workshop was finally attended by 57 people (health professionals with expertise in rare CD and rare NCDs from EU countries, Norway and from the EC) from 26 countries.

In the workshop, besides recognising the growing need to attend rare diseases related to mobility and globalization, the participants almost unanimously agreed to differentiate CD and NCD. The latter are already covered by different ERNs and should not be included in a new network. Concerning the benefit of a having a network on rare CD with a focus on Health Care provision, several arguments were raised:.

- To find spaces to further enable the discussion of clinical cases; and discussion between clinical and laboratory experts (e.g. in terms of test results, how to deal with false-positives etc.); and also as a meeting point of needs, feedbacks and expertise for Health Care Providers (HCPs) and the external environment (other networks, authorities, health systems, private sectors, etc.). This may reduce inappropriate practice variation.
- To promote the access to complex diagnostic tests and also to drugs for neglected diseases in order to reduce inequalities in health care.
- To give better advice to governments, or to be linking with the European regulatory bodies to improve access to orphan drugs; and to act as a promoter of harmonized strategies and actions (e.g. to improve the standards of screening strategies of imported diseases across Europe for blood or transplant donors, pregnant women or immunosuppressed individuals)
- To supervise and improve online available data and the registry of diseases that are not currently under surveillance, promoting the access to reliable information on new developments.
- To enhance translation of research into practice by reinforcing research and epidemiological surveillance and improve knowledge for physicians, healthcare providers, patients and families.
- To promote clinical evidence-based guidelines on imported diseases based on a robust methodology and exchange of healthcare professionals as well as training and education activities (e.g. webinars, e-learning courses or summer schools among others) would be a countless benefit.
- Cross-linking mechanisms with other ERNs should be promoted. Few examples of the types of collaborations in this regard include among others:

(i) To discuss and interact in some diseases that require a multidisciplinary approach (e.g. cysticercosis may require a follow-up from an Infectious diseases specialist but also from a Neurologist);
(ii) To discuss with experts belonging to other ERNs the specific problems and requirements of the growing number of travellers to tropical countries who are affected by chronic diseases, e.g. providing specific advice about the drug interaction with malaria chemoprophylaxis or about the contraindications of lived-attenuated vaccine.

3. The feasibility assessment was aimed at analysing how successful a European expert network on rare communicable diseases linked to mobility and globalization focused on Health Care provision would be based on the results of the review and the Consultation Workshop, considering which are the options for building the network.

The options and conditions of creating a European expert network for rare CD linked to Mobility and Globalisation (for diagnosis and treatment) were evaluated, considering three different scenarios:

1. “Status Quo” scenario, where current European networks identified would be enhanced but no new network would be built.
2. To create a specific European expert network (EEN) on rare CD linked to mobility and globalisation with the governance and structure to be defined.
3. To develop a new European Reference Network (ERN) on CD linked to mobility and globalisation in the same way as the current ERNs.

To do the assessment, the operational criteria of a potential network were developed and adapted from the criteria established by the European Commission for ERN (Operational Criteria for the Assessment of Networks) [26]. They were:

1. The establishment, including objectives and activities covered in the network, regulations of the network that should comply with EU regulations, but also the criteria and conditions to constitute the network.
2. The provision of highly specialized care in the areas of diagnosis, treatment and follow-up. This criterion included an assessment of the thematic areas and diseases covered by the network, the capability of improving health care provision and the capacity to create data registries,
3. The governance coordination and management, including the establishment of a board of the network, the coordinator, the criteria that newly formed HCP wishing to join an ERN must meet and the involvement of patients’ associations.
4. The ability to promote a good quality and safe patient care by fostering timely and pertinent diagnosis, treatment, follow-up and management across the Network.
5. Continuous education, training and development, including the creation or participation in evidence-based guidelines/recommendations, the sharing of knowledge and technologies and other training activities or education materials.
6. The capacity of conducting and collaborating in research activities (e.g. clinical trials) and the possibility of establishing shared registries and databases aiming to integrate existing resources.
7. The good practice, outcome measures and quality control measures for the network.
8. The multidisciplinary approach.
9. The capacity of establishing further networking and collaborations including the assessment of the added value that the network could bring to health care providers, as well as to other existing networks related to the area of expertise (e.g. other ERNs)
10. The funding sources including a management and business plan.
11. The evaluation of the sustainability considering the minimum of human resources required to maintain the network; possible future funding sources; how to ensure a roster and regular turnover of available experts to work on the network tasks in the future; and how will the information be made available to wider audiences (e.g. establishment of a website, organisation of seminars and conferences).

The detailed information about the results is summarized in Table 2. The main features of each scenario were:

1. “Status Quo” scenario: there are several existing networks with some focus on health care provision and patient care essentially improved through exchange of information, continuous education and training of health professionals. They are TROPNET, EUROTRAVNET and EVDLABNET. However, a proper system -according to EU data protection regulations- has not been implemented to share health data. There are some diseases (particularly endemic vector-borne diseases) not covered by these networks. All current networks are functioning with low financial resources, most of them private, and frequently informal and not necessarily recognised by the national health care systems.
2. A EEN would have the benefit of being created based on the findings and detected needs. Focus would be Health Care provision, and areas and diseases to be included would be the ones identified as rare diseases linked to mobility and globalisation. However, implementing a good system for data registry or discussion of clinical cases would be challenging in terms of sustainability. In addition, there is not a clear mechanism to guarantee the establishment of the EEN supported by the EC, and also the general networking and collaborations with external players would have to be developed. Finally, funding is needed and sources have to be identified, also in order to sustain the network, particularly if a special funding line is not put in place to support the new structure.
3. The basis and the legal status for an ERN have already been established. Governance is a complex aspect for ERNs [26]. Health Care Provision and specialized care status is decided by national health authorities and the participation and enrolment of centres are decided at national level [5]. The areas and diseases covered would be based on the detected needs. The current ERNs are supported with technological tools and online platforms facilitating the safe and ethically acceptable exchange of data, including the registries of cases. A call for a new ERN would provide the necessary funding to start the activity. Sustainability is a key aspect of the ERNs, although the experience is too recent to have a thorough model.

**Table 2:** Assessment of the three options for a potential network o rare Communicable diseases linked to Mobility and Globalisation

	Maintaining Status Quo	Creation of a European expert network (EEN)	Creation of a new ERN (ERN)	Other pending aspects to be explored
**Operational criteria**				
**Establishment of the network**	**+** There are three existing networks. There are members from non-EU/EEA countries in the networks. They comply the minimum of HCP and countries required. The main goal of the networks is not exclusively focused on Health care provision.	**+** The EC can set the basis but there are not currently clear mechanisms to be supported by EC (Health policy platform could be one). No clear mechanism on who should establish the expertise of the HCP. A minimum of HCP identified The HCP criteria could be more flexible compared with an ERN. It may partially overlap the focus of those existing networks.	**+ to ++** The EC and the Member States set the basis. The minimum of HCP identified. Health Care Provider status might make difficult the participation and enrolment of critical centres. It may partially overlap the focus of those existing networks.	The proposed number of required number of participants/ countries needs to be reviewed and validated
**Thematic areas to be covered by the network:**	**++** All networks are focused on Tropical and Travel related diseases which are rare communicable diseases linked to Mobility and Globalisation (one of them with an exclusively focus on viral infections).	**++** The network will be created based on the findings, and needs in terms of rare CD linked to Mobility and Globalisation.	**++** The network will be created based on the findings and needs in terms of rare CD linked to Mobility and Globalisation.	
**Disease or conditions covered**	**+ to ++** The existing networks already cover most of the diseases. In TropNet and EVDLabNet, there is not a list of diseases.	**++** The diseases to be included can be according to the detected needs. The list may not be a closed list since there are potential new public health threats for the EU/EEA countries in the future.	**++** The diseases to be included can be according to the detected needs. The list may not be a closed list since there are potential new public health threats for the EU/EEA countries in the future.	
**Improving the Health Care Provision**	**+** TropNet has a forum for discussion of clinical cases activity although ensuring the compliance with the EU data protection policy is currently a challenge. EVDLabNet has a directory of Laboratory capacities to improve the access to rare diagnostics tests.	**+** This criterion may be feasible depending on the legal basis and cross-exchange mechanisms of the network although ensuring compliance with the data EU protection policy may be challenging.	**++** This is the main guarantee of ERN, as well as its main goal, to ensure a better access to highly specialized healthcare.	
**Data registry**	**0 to +** TropNet and EuroTravNet do report number of cases yearly as a sort of surveillance activity but do not register clinical cases. The existing networks share data, but cannot be held responsible nor exchange personal patient data in a secure, safe and legally binding environment in compliance with the data EU protection policy, which is currently a challenge.	**+** It is difficult to assess the feasibility, as it will depend on the rules, data definition and legal framework of the network. This criterion may be feasible depending on the legal basis and cross-exchange mechanisms of the network although ensuring compliance with the EU data protection policy may be challenging.	**++** This is the main guarantee of ERN, as well as its main goal, to ensure a better access to highly specialized healthcare. ERNs register clinical cases for discussion and for a training purpose (CPMS system). In addition, another ERNs’ developments include online platform to safely and ethically share data, under the support of the EU in terms of technological tools enabling the sharing.	
**Governance, coordination and management of the network**	**0 to +** Existing networks have different governance and coordination structure. The networks might overlap in some aspects. It is currently foreseen to develop more collaborations. Members of the networks may not necessarily be HCP. Patients’ associations are not involved in these networks.	**+** A new network will mean new structures and roles, but it does not have to be too complex. It will depend on the organ (not necessarily the EC) to support the network. Patient’s associations could potentially be involved.	**++** The governance while complex is aspect of the ERNs has been already established. Patient’s associations could be potentially involved.	
**Patient care**	**+ to ++** The networks do not have guidelines to promote quality and safe patient care. Networks also provide information for patients/relatives within the network. EVDLabNet promotes the sharing of laboratory capabilities. TropNet has available a repository with information on orphan drugs available in the centres belonging to the network. No network has developed ICT-tools that could be developed to provide care, access to expertise, and support the development, sharing and spread of best practice. (E.g. Telemedicine, tele-expertise or remote consultation). Patient care is improved through continuous exchange of knowledge. Few centres from East European countries are involved in the networks.	**+ to ++** The specialization of the network should enable the creation of formally accepted professionalized guidelines for patient care. The involvement of HCP will depend on the Governance of the network Discussion of individual clinical cases could be proposed but the maintenance will be a challenge. ICT tools will depend on available funding. Most HCPs identified are from Western European countries. Efforts should be done to include more HCP from East European countries.	**++** The specialization of the network should enable the creation of formally accepted professionalized guidelines for patient care. One of the key function of the ERNs is to standardize care treatment, and the involvement of HCP directly connected to patients will ensure the direct translation of improved treatments. A forum for discussion of individual clinical cases will be proposed. ICT-tools could be developed to provide care, access to expertise, and support the development, sharing and spread of best practice. (E.g. Telemedicine, tele-expertise or remote consultation). Most HCPs identified are from Western European countries. Efforts should be done to include more HCP from East European countries. The network complies with the legal framework of the EC in providing Health Care.	The role and involvement of patients within the network needs to be clarified.
**Continuous education, training and development.**	**++** Activities related to continuous education and training are currently being conducted (seminar, scientific meeting, conferences...). They are very close to the professional needs, as facilitated by the exchange and informal atmosphere.	**+** These activities would be very close to the professionals needs, and targeted to a very specific field. Depending on the resources available, they will be implemented more easily.	**++** These activities would very close to the professionals needs, and facilitated by the exchange of information on the official online platforms. Patients’ education material are promoted by ERN.	Training and educational activities are limited or not for members of the network.
**Research activities of the network.**	**++** The health professionals already contribute to research by developing R&I projects together, including clinical trials, (particularly TropNet).	**?** The creation of the network itself is part of an R&I process. The founders will need to decide the relevance of the research for the activities of the network.	**+ to ++** The main objective of the ERNs is to improve healthcare services, and the research activities, although secondary, are encouraged through the consortium, and the individuals. Time is a constraint for clinicians involved.	If external funding to fund research is needed. The amount of time to be dedicated for research by clinicians
**Good practice, outcome measures and quality control measures for the network**	**0 to +** Although the networks promote the exchange of good practices, it is not mandatory to follow up and monitor the concrete impact of the exchanges, and the uptake of new/improved guidelines. Cross-border collaborations to improve the health care provision have been already implemented (e.g. EVDLabNet laboratory capacity sharing or TROPNET – information access to Orphan drugs). There is not a defined quality control planning with clear objectives and measurable indicators for monitoring and evaluation, particularly regarding the compliance of the access to medical records and clinical information with the EU regulations.	**+** Through the formalisation of the network, a set of outcome measures and monitoring should be set, and agreed by the founders, especially to justify costs and impact, and obtain funding for their activities. However, to assure the compliance of the access to medical records and clinical information with the EU regulations will be challenge to create and maintain a system to assure the confidentiality. The network could also create a standard procedure for obtaining consent form.	**++** ERNs are monitored and controlled by both the HCP and the funder (DG-Sante). A series of Key Performance Indicators (KPI) have to be established, and their outcomes and impact is measured. ERN requires a quality control planning with clear objectives and measurable indicators for monitoring and evaluation, particularly regarding the compliance of the access to medical records and clinical information with the EU regulations. The network would also create a standard procedure for obtaining consent form.	Define the set of Key Performance Indicators to ensure monitoring and impact driven approach.
**Multidisciplinary approach**	**+** The existing networks have a very open approach towards multidisciplinary collaboration, particularly EuroTravNet. No clear rules within the networks on the openness towards other disciplines is established.	**?** The founders will need to decide the relevance given to multi/trans-disciplinary approach, within the scheme decided and the concrete objectives/goals of the network.	**++** ERN will ensure a multidisciplinary approach to treat the diseases/ affectations at the core of the network. There is also 24 other ERNs, which meet and exchange at least on a bi-annual basis, summing up to the concrete specificities of each one of them.	Explore how to add social and gender approach to the different networks.
**Networking and collaboration**	**+ to ++** Networks are already established, and although collaboration is well engaged, both within and towards other networks, it is necessary to establish it specifically on Health Care Provision. No official collaborations with other disciplines have been established (blood banks, transplant or antenatal care units among other).	**+** Although based on existing partnership, and related centres, the general networking and collaborations towards external players would have to be developed.	**+ to ++** The general networking and collaborations towards external players would have to be developed. However, collaboration with other ERNs may be facilitated through the ERN structure.	Define better priority lines of collaboration.
**Funding and sustainability criteria**				
**Funding sources**	**0 to +** Networks are already functioning, although with low or non - financial resources. EVDLabNet is funded by the ECDC. Private funding sources: EuroTravNet is currently funded by private institutions (International Society of Travel Medicine and the Instituts Hospitalo-Universitaire Méditerranée Infection Foundation, Marseille). TROPNET is not funded.	**?** Important funding is needed to create a network. The feasibility is very much depending if a special funding line is put in place to support the new structure or if the sources have to be identified and obtained through standard competitive processes.	**++** To start the activities, an ERN could receive resources from EU Health programme. A call for a new ERN would provide the necessary funding to start the activity.	Funding sources need to be available in order to start new activities, or consolidate those already existing.
**Sustainability analysis of the network**	**0 to +** For now, networks have been sustainable, as shown on their existence overtime, and some key collaborations or merger are sought for. It is unclear though how sustainability is foreseen over time if networks wish to grow and to strengthen the activities focused on Health Care Provision.	**?** To ensure sustainability should be part of the basis of the new network. If there is a possibility to get the funds, a sustainability strategy should be put in place, through the engagement of the partners.	**+ to ++** Sustainability is a key aspect of the ERNs, although for now, the experience is too recent to have a concrete model (publicly funded, through European or National schemes) that can be replicated for this new ERN. It is unclear yet how they will become self-sustainable overtime.	Define the basis of self-sustained network, ensure sustainability through multiple funders, but also through clear and set engagement of the stakeholders involved.

Full note: In the assessment of each scenario, each criterion was rated once for the overall network. Each criterion has been graded in the following way: “0” The scenario proposed does not fulfil the criterion; “+” The scenario proposed fulfil the criterion with limitations; “++” The scenario proposed fulfils the criterion; “?” Difficult to assess, (for the variables and knowledge limitations on the aspects assessed)

## Discussion

Providing highly specialised diagnosis, treatment and care for patients who have complex diseases can be a challenge [27]. This is especially true when the prevalence of such diseases is low, or when a new disease is emerging as is the case for rare diseases linked to mobility and globalisation. This challenge is due both to the scarcity of expertise and to the scattering of small patient populations across the EU, sometimes in isolated locations where expertise does not exist or cannot be accessed. As a way of example, the evolution of the COVID-19 pandemic is affecting the European Member States (EU MS) with different levels of severity and with an epidemiological situation is evolving very quickly [28]. Although it is not a rare disease anymore in Europe, and the level of knowledge is similar in all EU countries, it is a novel emerging disease unknown for most health professionals and to establish a proper system for sharing information and to establish potential collaborations across countries would be highly valuable.

Many patients are in the need of highly specialised care in third level hospitals. The practical experience on how to manage the patients and in particular the severe cases is scarce and scattered in Europe [27]. While the experience and the number of cases treated by some Hospitals (and MS) is important, due the accumulated expertise, other are still starting to deal with complex patients. Following the previous example, the treatments applied to the COVID-19 patients are in many cases experimental and there are some limitations to reach the knowledge generated during the last months and weeks. Therefore, a new ERN could help to integrate the expertise and competence of those that have already have them with the healthcare professionals that are directly in charge of the patients by facilitating a quick exchange of knowledge and experiences and assisting in the decision making process and ultimately to benefit the patients suffering the condition [27].

A European network on rare CD linked to mobility and globalisation may provide a unique opportunity for clinicians to work across borders in EU/EEA countries in healthcare provision in order to tackle this challenge. Coordination strategies among EU/EEA countries to fight against new emerging communicable diseases may be facilitated if there is a network oriented to improve the diagnosis capacity and treatment options for patient and learning from health professional experiences from EU/EEA countries where the diseases have progressed first. The network could also be well aligned with other European initiatives or programmes in place on communicable diseases such as the ECDC associated networks, or the One Health European Joint Programme without overlapping or eclipsing any function or role of such programmes or initiatives.

On the other hand, the study has several limitations that need to be recognized. First, the heterogeneity of the access to health care provision for individuals at risk of mobility associated infectious diseases across EU countries has not been properly evaluated. This is of particular importance for undocumented migrants who have restrictive access to health care services. This element needs to be considered and evaluated when the network is implemented. Second, the connection with other initiatives or programmes with common objectives at national and also at EU level need to be better evaluated to be complementary instead of overlapping activities. Finally, the sustainability of is a key aspect as per the rest of ERN that need a widespread assessment although for now, the experience of ERNs is too recent to have a concrete model.

## Conclusions

If the focus is the health care provision, it could be concluded that an ERN could have the potential to better boost the quality of care. However, current existing networks should not be eclipsed by a potential new network and they should be complementary. Future steps should explore the Member states view and discussion on the appropriateness for launching a new ERN.

## Supplementary information

**Additional file 1.**

## Data Availability

Supporting data will be available through request to the EC who are the owners of the data.

## Abbreviations

EU: European Union
EEA: European Economic Area
ERN: European Reference Network
EC: European Commission
CD: Communicable diseases
NCD: Non communicable diseases
SCD: Sickle cell disease
HCP: Health Care Providers

## References

[1] Gushulak B, Weekers J, Macpherson D (2009). Migrants and emerging public health issues in a globalized world: threats, risks and challenges, an evidencebased framework. Emerg Health Threats J.

[2] European Centre for Disease Prevention and Control. Migrant health: Background note to the ‘ECDC Report on migration and infectious diseases in the EU’: ECDC; 2009.

[3] European Commission. Rare diseases from: https://ec.europa.eu/health/rare_diseases/policy_en. Accessed 22 Sept 2020.

[4] European Commission (2008). Communication from the commission to the European Parliament, the council, the European economic and social committee and the committee of the regions.

[5] Héon-Klin V (2017). European reference networks for rare diseases: what is the conceptual framework?. Orphanet J Rare Dis.

[6] Hartman AL, Hechtelt Jonker A, Parisi MA, Julkowska D, Lockhart N, Isasi R (2020). Ethical, legal, and social issues (ELSI) in rare diseases: a landscape analysis from funders. Eur J Hum Genet 2019.

[7] Perez-Molina JA, Lopez-Polin A, Trevino B, Molina I, Goikoetxea J, Diaz-Menendez M, et al. 6-year review of +Redivi: a prospective registry of imported infectious diseases in Spain. J Travel Med. 2017;24(5). 10.1093/jtm/tax035 28931128

[8] Abubakar I, Aldridge RW, Devakumar D, Orcutt M, Burns R, Barreto ML (2018). The UCL-Lancet Commission on migration and health: the health of a world on the move. Lancet..

[9] Lindenmeyer A, Redwood S, Griffith L, Teladia Z, Phillimore J (2016). Experiences of primary care professionals providing healthcare to recently arrived migrants: a qualitative study. BMJ Open.

[10] European Centre for Disease Prevention and Control. Assessing the burden of key infectious diseases affecting migrant populations in the EU/EEA: ECDC; 2014. Contract No.: TQ-04-14-474-EN-N.

[11] Bruckner GK (2011). Ensuring safe international trade: how are the roles and responsibilities evolving and what will the situation be in ten years’ time?. Rev Sci Tech.

[12] Medlock JM, Hansford KM, Versteirt V, Cull B, Kampen H, Fontenille D (2015). An entomological review of invasive mosquitoes in Europe. Bull Entomol Res.

[13] Medlock JM, Leach SA (2015). Effect of climate change on vector-borne disease risk in the UK. Lancet Infect Dis.

[14] Le Tyrant M, Bley D, Leport C, Alfandari S, Guegan JF (2019). Low to medium-low risk perception for dengue, chikungunya and Zika outbreaks by infectious diseases physicians in France, Western Europe. BMC Public Health.

[15] Mathis M, Briand S (2019). Climate change, epidemics and the importance of travel and tropical medicine. Rev Med Suisse.

[16] Mancuso JD, Garges EC, Hickey PW, Coldren RL, Korman AK, Keep LW (2017). Outcomes from U.S. military-supported overseas training rotations in tropical medicine and Global Health, 2006-2015. Mil Med.

[17] Hoffmann T, Straus S (2017). Sharing knowledge for health care. JAMA Intern Med.

[18] Zur B (2016). Increase in genetically determined anemia as a result of migration in Germany. Internist (Berl).

[19] Gelpi AP (1973). Migrant populations and the diffusion of the sickle-cell gene. Ann Intern Med.

[20] European Commission (2019). European Reference Networks: European Commission.

[21] Orphanet list of rare diseases. https://www.orpha.net/consor/cgi-bin/Disease_Search_List.php?lng=EN. Accessed 20 Mar 2020.

[22] European Network for Tropical Medicine and Travel Health – TROPNET. http://tropnet.eu/. Accessed 21 Mar 2020.

[23] The European Travel and Tropical Medicine Network of the Internatinal Society of Travel Medicine (EuroTravNet). https://www.istm.org/eurotravnet. Accessed 21 Mar 2020.

[24] The European Expert laboratory network for emerging viral diseases (EVD-LabNet). https://www.evd-labnet.eu/. Accessed 21 Mar 2020.

[25] Prospero Registry - Rare communicable diseases in Europe linked to mobility and globalization: a protocol for a systematic review and meta-analysis of disease frequencies. https://www.crd.york.ac.uk/prospero/display_record.php?RecordID=126549. Accessed 22 Sept 2020.

[26] Heon-Klin V. European reference networks for rare diseases: what is the conceptual framework? Orphanet J Rare Dis. 2017;12(1):137. 10.1186/s13023-017-0676-3 PMC5547471 28784158

[27] Wijnen R, Anzelewicz SM, Petersen C, Czauderna P (2017). European reference networks: share, care, and cure-future or dream?. Eur J Pediatr Surg.

[28] Bade LR, Rubin EJ (2020). Covid-19. The search for effective therapy. N Engl J Med.

