# Supplementary material for: European expert network on rare communicable diseases and other rare diseases linked to mobility and globalisation focused on health care provision (EURaDMoG): a feasibility study
Source: Orphanet J Rare Dis. 2020 Oct 16;15:291. doi: 10.1186/s13023-020-01534-1 (PMC7563907; doi:10.1186/s13023-020-01534-1)
Supplement: Supplementary file 1 — Additional file 1. [file 13023_2020_1534_MOESM1_ESM.pdf]

ANNEX 1. LIST OF RARE COMMUNICABLE DISEASES LINKED TO MOBILITY AND GLOBALISATION IDENTIFIED THROUGHOUT THE EURADMOG STUDY.

RARE COMMUNICABLE DISEASES RELATED TO MOBILITY AND GLOBALISATION

DISEASE related keywords	MICROORGANISM related keywords
**BACTERIAL INFECTIONS**	
Anaplasmosis	*Anaplasma phagocytophilum*
Ehrlichiosis	*Ehrlichia* spp.
Endemic typhus, Murine typhus /flea-borne typhus	*Rickettsia typhi*
Epidemic typhus	*Rickettsia prowazekii*
Mediterranean spotted fever	*Rickettsia conorii*
Rickettsialpox	*Rickettsia akari*
Scrub typhus, Tsutsugamushi disease	*Orientia tsutsugamushi*
Rocky mountain spotted fever	*Rickettsia rickettsii*
African tick typhus	*Rickettsia africae*
Relapsing fever	*Borrelia recurrentis*
Lyme disease	*Borrelia burgdoferi*
Other rickettsiosis	*Rickettsia* spp.
Anthrax	*Bacillus anthracis*
Tetanus	*Clostridium tetani*
Trachoma	*Chlamydia trachomatis*
Chancroid	*Haemophilus ducreyi*
Granuloma inguinale- Donovanosis	*Klebsiella granulomatis*
Actinomycosis	*Actinomyces israelii*

Treponema infections Yaws	*Treponema pallidum pertenue*
Buruli ulcer	*Mycobacterium ulcerans*
Hansen's disease / Leprosy	*Mycobacterium leprae*
Leptospirosis	*Leptospira interrogans*
Bartonellosis, Oroya fever, Carrion disease	*Bartonella bacilliformis*
Bartonellosis, Trenchs fever	*Bartonella quintana*
Brucellosis, Malta fever	*Brucella* spp.
Melioidosis	*Burkholderia pseudomallei*
Paratyphoid fever	*Salmonella enterica* serotype Paratyphi
Typhoid fever	*Salmonella enterica* serotype Typhi
Rheumatic fever	*Streptococcus pyogenes*
Q fever, Nine mile fever, Quadrilateral fever, Query fever	*Coxiella burnetii*
Secondary non-tropical sprue - Whipple disease	*Tropheryma whipplei*
Cholera	*Vibrio cholerae*
Botulism	*Clostridium botulinum*
Tropical pyomyositis	---
Tularemia	*Francisella tularensis*
Plague	*Yersinia pestis*
Diphtheria	*Corynebacterium diphtheriae*

FUNGAL INFECTIONS

Chromomycosis/Chromoblastomycosis	*Fonsecaea pedrosoi, Phialophora verrucosa and Cladophialophora carrionii*
Madura foot /Eumycetoma	*Madurella mycetomatis*
Sporotrichosis	*Sporothrix schenckii*

Paracoccidioidomycosis	*Paracoccidioides brasiliensis*
Coccidioidomycosis, Desert fever, San Joaquin valley fever, California disease	*Coccidioides immitis*
Peniciliosis	*Penicillium marneffei*
Scedosporiosis	*Scedosporium* spp.
**HELMINTH INFECTIONS**	
Cystic echinococcosis, Echinococcosis	*Echinococcus granulosus*
Alveolar echinococcosis	*Echinococcus multilocularis*
Ancylostomiasis /Ankylostomiasis	*Ancylostoma duodenale /Necator americanus*
Angiostrongyliasis	*Angiostrongylus cantonensis*
Strongyloidiasis, Anguilluliasis	*Strongyloides stercoralis*
Anisakiasis	*Anisakis* spp.
Ascariasis	*Ascaris lumbricoides*
Bilharziasis, Schistosomiasis	*Schistosoma* spp.
Clonorchiasis	*Clonorchis sinensis*
Opisthorchiasis	*Opisthorchis viverrini*
Cutaneous larva migrans	*Ancylostoma* spp.
Cysticercosis	*Taenia solium*
Diphyllobothriasis	*Diphyllobothrium latum*
Dirofilariasis	*Dirofilaria immitis*
Distomatosis	*Other trematodes-Heterophyes heterophyes , Metagonimus* spp.
Dracunculiasis, Guinea Worm disease	*Dracunculus medinensis*
Fascioliasis	*Fasciola* spp.
Paragonimiasis	*Paragonimus* spp.
Gnathostomiasis	*Gnathostoma* spp.

Hymenolepiasis	*Hymenolepsis nana*
Loiasis	*Loa loa*
Lymphatic filariasis	*Wuchereria bancrofti / Brugia malayi*
Mansonelliasis	*Mansonella* spp.
Onchocerciasis	*Onchocerca volvulus*
Sparganosis	*Spirometra* spp.
Taeniasis	*Taenia* spp.
Trichinosis	*Trichinella spiralis*
Trichuriasis	*Trichuris trichiura*

PROTOZOAL INFECTIONS

Acanthamoeba infection, Keratitis	*Acanthamoeba* spp.
African trypanosomiasis, Sleeping sickness	*Trypanosoma brucei*
Amebiasis	*Entamoeba histolytica*
Amebic meningoencephalitis	*Naegleria fowleri*
American trypanosomiasis, Chagas disease	*Trypanosoma cruzi*
Babesiosis	*Babesia* spp.
Cryptosporidiosis	*Cryptosporidium parvum*
Cyclosporiasis	*Cyclospora cayetanensis*
Isosporiasis	*Isospora belli*
Leishmaniasis	*Leishmania* spp.
Malaria	*Plasmodium* spp.
Sarcocystosis, Sarcosporidiosis	*Sarcocystis hominis*

VIRAL INFECTIONS

Avian flu	Avian influenza
Brazilian haemorrhagic fever	Sabia virus
California encephalitis	California encephalitis virus
Chapare haemorrhagic fever	Chapare virus
Chikungunya	Chikungunya virus
Colorado tick-borne disease, Mountain fever, American mountain fever, Mountain tick fever-	Colorado tick fever (CTF)
Crimea-Congo haemorrhagic fever	CCHF virus
Dengue fever	Dengue virus
Ebola	Ebola virus
Hantavirosis, Haemorrhagic fever-renal syndrome	Hantavirus
Hepatitis D	Hepatitis D virus
Hepatitis E	Hepatitis E virus
Herpes B infection, B virus infection	Herpesvirus simiae, monkey B virus
Tropical spastic paraparesis	Human T-lymphotropic virus 1 (HTLV-1)
Japanese encephalitis	Japanese encephalitis virus (JEV)
Junin haemorrhagic fever, Argentine haemorrhagic fever	Junin virus
Kyasanur haemorrhagic fever, Kyasanur forest disease, Monkey fever, Monkey disease	KFD virus (KFDV)
La Crosse encephalitis	La Crosse virus (LACV)
Lassa haemorrhagic fever	Lassa virus
Lujo haemorrhagic fever, Zambian haemorrhagic fever	Lujo virus

Machupo haemorrhagic fever	Machupo virus (MACV)
Marburg haemorrhagic fever /Marburg virus disease	Marburg virus
Nipah encephalitis, Nipah fever /Nipah virus disease	Nipah virus
Omsk haemorrhagic fever	Omsk haemorrhagic fever virus (OHFV)
Poliomyelitis, Poliomyelitis in patients with immunodeficiency deemed at risk	Poliovirus
Rabies	Rabies virus
Rift valley fever	Rift valley fever (RVF) virus
Saint Louis encephalitis	Saint Louis encephalitis (SLE) virus
Tick-borne encephalitis	Tick-borne encephalitis (TBE) virus
Venezuelan haemorrhagic fever	Guanarito virus
Western equine encephalitis, Western equine encephalomyelitis	Western equine encephalitis (WEE) virus
West-Nile encephalitis, West-Nile fever	West-Nile virus (WNV)
Yellow fever	Yellow fever virus
Zika virus disease	Zika virus
Middle East respiratory syndrome coronavirus	MERS-CoV
**OTHER CONDITIONS**	
Tick paralysis	Tick
Cutaneous myiasis	*Dermatobia hominis, Cordylobia anthropophaga*
African iron overload	
Ciguatera fish poisoning	
Tropical calcific chronic pancreatitis	Tropical pancreatitis
Tropical pancreatic diabetes	

Tropical endomyocardial fibrosis (TEF)	
Acquired Creutzfeldt-Jakob disease	prion disease
Hyperreactive malarial splenomegaly	
Genital female mutilation	
Severe fever with thrombocytopenia syndrome	
**INFECTIONS WITH PARTICULAR ASPECTS TO BE CONSIDERED**	
Giardiasis	*Giardia duodenalis*
Shiga-like toxin-associated HUS	*E.coli* (O157)
Tuberculosis	*Mycobacterium tuberculosis*
